# Bubaline Cholecyst Derived Extracellular Matrix for Reconstruction of Full Thickness Skin Wounds in Rats

**DOI:** 10.1155/2016/2638371

**Published:** 2016-04-05

**Authors:** Poonam Shakya, A. K. Sharma, Naveen Kumar, Remya Vellachi, Dayamon D. Mathew, Prasoon Dubey, Kiranjeet Singh, Sonal Shrivastava, Sameer Shrivastava, S. K. Maiti, Anwarul Hasan, K. P. Singh

**Affiliations:** ^1^Division of Surgery, Indian Veterinary Research Institute, Izatnagar, Uttar Pradesh 243 122, India; ^2^Division of Veterinary Biotechnology, Indian Veterinary Research Institute, Izatnagar, Uttar Pradesh 243 122, India; ^3^Centre for Animal Disease Research and Diagnosis, Indian Veterinary Research Institute, Izatnagar, Uttar Pradesh 243 122, India

## Abstract

An acellular cholecyst derived extracellular matrix (b-CEM) of bubaline origin was prepared using anionic biological detergent. Healing potential of b-CEM was compared with commercially available collagen sheet (b-CS) and open wound (C) in full thickness skin wounds in rats. Thirty-six clinically healthy adult Sprague Dawley rats of either sex were randomly divided into three equal groups. Under general anesthesia, a full thickness skin wound (20 × 20 mm^2^) was created on the dorsum of each rat. The defect in group I was kept as open wound and was taken as control. In group II, the defect was repaired with commercially available collagen sheet (b-CS). In group III, the defect was repaired with cholecyst derived extracellular matrix of bovine origin (b-CEM). Planimetry, wound contracture, and immunological and histological observations were carried out to evaluate healing process. Significantly (*P* < 0.05) increased wound contraction was observed in b-CEM (III) as compared to control (I) and b-CS (II) on day 21. Histologically, improved epithelization, neovascularization, fibroplasia, and best arranged collagen fibers were observed in b-CEM (III) as early as on postimplantation day 21. These findings indicate that b-CEM have potential for biomedical applications for full thickness skin wound repair in rats.

## 1. Introduction

Skin protects the body from the external environment by maintaining temperature and homeostasis, as well as by performing immune surveillance and sensory detection [1]. Significant skin loss due to injury, genetic disorders, acute trauma, chronic wounds, or surgical procedures leading to damage of dermal or subdermal tissues cannot heal properly and can lead to serious consequences. Most wounds can heal naturally, but full thickness wounds greater than 1 cm in diameter need a skin graft to prevent scar formation, resulting in impaired morbidity and cosmetic deformities [2].

Biological scaffolds derived from decellularized tissues are in use as surgical implants and scaffolds for regenerative medicine because extracellular matrix secreted from resident cells of each tissue and organ can provide favorable microenvironment that affects cell migration, proliferation, and differentiation [3, 4]. The biomaterials are materials intended to interface with biological systems to evaluate, treat, augment, or replace any tissue, organ, or function of the body [5]. Use of the acellular dermal graft in abdominal wall defects was reported with good success rate in rabbits [6]. Acellular biomaterials can stimulate the local environment to repair tissues without the regulatory and scientific challenges of cell-based therapies [7].

It is obvious that a scaffold mimicking the extracellular matrix (ECM) with adequate bioactive molecules, capable of supporting the growth of cells participating in regeneration, is an ideal graft suitable for wound healing application [8]. Indeed, ECM isolated from certain mammalian organs and tissues have been found to have these essential biocomponents that support cell proliferation, migration, and differentiation [9]. These scaffolds are naturally rich in collagen, elastin, glycosaminoglycans (GAGs), laminin, and fibronectin on which the cells can migrate, attach, and grow. In addition, many of the bioactive degradation products released from the graft at the site of the grafting mimic growth factors required for healing [10]. The ECM is also known to aid in angiogenesis by regulating the migration, proliferation, and sustenance of endothelial cells [11]. Hence, ECM is correctly termed as nature's ideal scaffold material [12].

The small intestinal submucosa (SIS) is a popular and favorite scaffold that helps in constructive remodelling of injured sites in many parts of the body, including chronic wounds such as nonhealing leg ulcers [13]. However, some studies also reported that the SIS have no significant beneficial effect in treating full thickness skin wounds in dogs [14] but may elicit some adverse inflammatory response [15]. There are also reports of complications arising from the clinical use of small intestine submucosa in other regenerative medical applications [16]. Hence, there is a quest for better biological scaffolds for regenerative medical applications in general and skin graft substitutes in particular.

Cholecyst derived extracellular matrix (CEM) recovered from ECM of porcine gallbladder had variable application in the field of regenerative medicine [17]. The cholecyst derived ECM is shown to act as reinforcing buttressing staples for gastrointestinal resection and anastomotic procedures [18]. It has a microarchitecture similar to heart valves, supports proliferation of valvular endothelial and interstitial cells [19], and has ability to withstand large strain [20]. This cholecyst derived extracellular matrix (CEM) is found to be a novel acellular proteinaceous biodegradable biomaterial and may have potential applications as scaffolds in heart valve tissue engineering. This matrix is rich in collagen and contains several other macromolecules useful in tissue remodelling [20]. Burugapalli et al. [21] reported that CEM allowed complete ingrowth of cells as well as production of natural extracellular matrix (ECM), when implanted subcutaneously in rat model. Burugapalli et al. [22] used different cross-linking agents as a tool for tailoring the properties of cholecyst derived extracellular matrix. It was found that EDC cross-linking was successfully used to tailor the biological stability of CEM.

Keeping in view the above-mentioned facts, the present study was undertaken to test the efficacy of bovine cholecyst derived acellular matrix (b-CEM) for the repair of full thickness skin defects in a rat model.

## 2. Materials and Methods

Standard reagents were obtained from Sigma-Aldrich (St. Louis, MO, USA) unless otherwise noted.

### 2.1. Preparation of Acellular Bovine Gallbladder Matrix

Gallbladder of bovine was collected from local abattoir and kept in cold normal saline solution containing 0.02% EDTA and antibiotic (amikacin 1 mg/mL). The tissue was rinsed with normal saline before the start of the protocol. The maximum time period between retrieval and initiation of protocol was less than 4 hours. The neck and fundus of the gallbladder were trimmed, followed by a longitudinal incision to obtain a flat sheet of tissue. The inner mucosal layer was peeled off and the outer serosal layer was removed by mechanical delamination with a blunt edge. The tissue was cut into 2 × 2 cm^2^ size pieces and treated with 70% ethanol for 4 h and later on with distilled water for 24 h. The gallbladder tissue was decellularized using sodium dodecyl sulphate biological detergent in 0.5% concentration in 10 mM Tris buffer solution for 24 to 72 h. The acellularity and collagen fiber arrangement were assessed at every 12 h interval by histopathological examination. The tissues were thoroughly washed in phosphate buffered saline (PBS) solution and were stored at 4°C in PBS solution containing 0.1% amikacin till further use.

### 2.2. Animals and Ethics Statement

Protocols for this study were approved by the Institute Animal Ethics Committee of the Indian Veterinary Research Institute (IVRI), Izatnagar, Uttar Pradesh, India, according to the guidelines for the care and use of laboratory animals. All efforts were made to minimize the animal suffering and reduce the number of animals used. Thirty-six clinically healthy Sprague Dawley rats of either sex, weighing from 250 to 300 g, and of 3 to 4 months of age were used in this study. Rats were procured from the Laboratory Animals Resource Section of the Animal Genetics Division at the IVRI. Animals were housed individually in cages, provided with commercial diet and water ad libitum in a temperature and humidity-controlled environment. The animals were acclimatized to approaching and handling for a period of 10 days before the start of study.

### 2.3. Skin Wound Creation and Implantation

Animals were anesthetized using xylazine (5 mg/kg body weight) and ketamine (50 mg/kg body weight) combination [23]. The animals were restrained in sternal recumbency and dorsal thoracic area was prepared for aseptic surgery. Using a sterile plastic template the vertices of the experimental wounds of 20 × 20 mm^2^ dimensions were outlined on the dorsothoracic region of the rats. A full thickness skin defect including the* panniculus carnosus* was excised with a #11 BP blade on each animal. Haemorrhage, if any, was controlled by applying pressure with sterile cotton gauze. The defect in group I (C) was kept as open wound and was taken as control (C). In group II, the defect was repaired with commercially available collagen sheet (b-CS) (Skin Temp*™* II, Absorbable Bovine Collagen, Polyethylene Non-Woven Mesh, Human BioSciences India Limited, 142/143P Vasna Chacharwadi, Ahmedabad 382213, India). In group III, the defect was repaired with cholecyst derived extracellular matrix of bubaline origin (b-CEM). The scaffolds were secured to the edge of the skin wounds with eight simple interrupted 4/0 nylon sutures. The repaired wounds were covered with paraffin surgical dressings. After recovery from anesthesia, rats were housed individually in properly disinfected cages. Further, antimicrobial treatment (enrofloxacin 10 mg/kg intramuscularly once a day) was continued for five days and meloxicam (0.3 mg/kg intramuscularly once a day) for three days.

### 2.4. Evaluation of Wound Healing

#### 2.4.1. Wound Contraction

Wound area of all groups was measured by tracing its contour using transparent sheets with graph paper on postoperative days 0, 3, 7, 14, 21, and 28. The area (mm^2^) within the boundaries of each tracing was determined and percent contraction was measured by a rate of wound reduction, as well as percent reduction in wound area.

#### 2.4.2. Planimetry

Colour photographs were taken on postimplantation days 7, 14, 21, and 28 with the help of digital camera at a fixed distance. Analysis of shape, irregularity, and colour of the lesion were determined.

#### 2.4.3. Haematological Observations

Blood smears for total leukocyte count (TLC) and differential leukocyte count (DLC) were prepared on postoperative days 0, 3, 7, 14, 21, and 28. The blood smears were stained with Leishman stain for 15–20 minutes [24]. The counts were expressed in percent.

#### 2.4.4. Immunological Observations

*(1) Humoral Response*. For the ELISA procedure serum was collected on days 0 and 28 from each rat. Harvested sera were assessed for the extent of antibodies titer generated toward the implant. Briefly, 96-well flat bottom polystyrene plate was coated with 0.25 mg protein (derived from implant material) in 100 *μ*L/well of 0.05 M sodium carbonate/sodium bicarbonate coating buffer (pH 9.6) per well. The plate was covered with aluminum foil and incubated at 4°C overnight and washed 4 times using washing buffer (PBS with 0.05% Tween-20; PBS-T; pH 7.4). The wells were blocked with 5% skimmed milk powder (200 *μ*L/well) for 3 h and washed 4 times. All plate washes and sample dilutions were made with PBS-T. Serum samples were run in triplicate. One hundred microlitre (100 *μ*L) of diluted (1 : 100) test serum was added to each of antigen-coated well and incubated for 2 h at 37°C. The plate was washed and reincubated for 2 h with 100 *μ*L/well of the secondary antibody (goat anti-rat immunoglobulin G conjugated with horseradish peroxidase) in a 1 : 30,000 dilution. The plate was washed 4 times prior to the addition of 100 mL/well of the freshly prepared enzyme substrate solution (citric acid 0.1%, Na3PO4 0.1 M, 0.4 mg/mL* o*-phenylenediamine, and 0.03% hydrogen peroxide) and was incubated for 15 min at 37°C and stopped with 3 M solution of sulfuric acid. Absorbance (OD) values were recorded at 492 nm wavelength using an automated ELISA plate reader (ECIL, Hyderabad, India). The antibodies titer present in serum samples harvested on day zero was taken as basal values.

*(2) Cell Mediated Immune Response*. The cell mediated immune response was assessed by 3-(4,5-dimethylthiazol-2-yl)-2,5-diphenyltetrazolium bromide (MTT) colorimetric assay. The stimulation of rat lymphocytes with phytohemagglutinin (PHA) was considered as positive control, whereas unstimulated culture cells were taken as negative control. Blood collected from rats with implants (b-CS and b-CEM) on postimplantation days 0 and 28 was used for lymphocyte culture as per standard method. Briefly, 1 mL of blood was aseptically collected in a heparinized tube and mixed with equal volume of 1xPBS. It was carefully layered over 2 mL of lymphocyte separation medium (Histopaque 1077) and centrifuged at 2000 rpm for 30 min. The buffy coat was harvested in a fresh tube and two washings were done with 1xPBS at 1000 rpm for 5 min. Supernatant was discarded and pellet was resuspended in RPMI 1640 growth medium supplemented with 10% fetal bovine serum. The cells were adjusted to a concentration of 2 × 10^6^ viable cells/mL in RPMI 1640 growth medium and seeded in 96-well tissue culture plate (100 *μ*L/well). The cells were incubated at 37°C in 5% CO_2_ environment. Cells from each rat were stimulated with antigens (b-CS and b-CEM) (10–20 *μ*g/mL) and PHA (10 *μ*g/mL), a T-cell mitogen, in triplicate and three wells were left unstimulated for each sample. After 45 h, 40 *μ*L of MTT solution (5 mg/mL) was added to all the wells and incubated further for 4 h. The plates were then centrifuged for 15 min in plate centrifuge at 2500 rpm. The supernatant was discarded, plates were dried, and 150 mL dimethyl sulfoxide was added to each well and mixed thoroughly by repeated pipetting to dissolve the formazan crystals. The plates were immediately read at 570 nm with 620 nm as reference wavelength. The stimulation index (SI) was calculated using the formula SI = OD of stimulated cultures/OD of unstimulated cultures.

#### 2.4.5. Histological Evaluation

The biopsy specimen from the implantation site was collected on 3, 7, 14, 21, and 28 postimplantation days for histopathological evaluation. Biopsy samples were fixed in 10% formalin saline. The samples were then processed for paraffin embedding technique to get 5 micron thick paraffin sections. The sections were stained by hematoxylin and eosin as per standard protocol. The host inflammatory response, neovascular tissue formation (fibroblasts, fine capillaries), deposition of neocollagen, and penetration of host inflammatory responses in the implanted matrix were evaluated as per Schallberger et al. [14]. Special staining for collagen fibers was done by using Masson's trichrome stain [25] to observe the deposition of collagen fibers and scoring was done as per the method of Borena et al. [26]. The histological parameters were graded as follows: epithelization: 1—present, 2—partially present, and 3—absent; inflammation: 1—resembling normal skin, 2—mild, 3—moderate, and 4—severe; fibroplasia: 1—resembling normal skin, 2—mild, 3—moderate, and 4—severe; neovascularization: 1—resembling normal skin (0-1 new blood vessels), 2—mild (2–5), 3—moderate (6–10), and 4—severe (>10); collagen fiber density: 1—denser, 2—dense, and 3—less dense; collagen fiber thickness: 1—thicker, 2—thick, and 3—thin; and collagen fiber arrangement: 1—best arranged, 2—better arranged, 3—badly arranged, and 4—worst arranged. The group having less histopathological score was considered the best.

### 2.5. Data Analysis

Results were expressed as mean ± standard error of mean (SE). Data were analyzed by one-way variance (ANOVA). The means were compared by Tukey's test for multiple comparisons, with *P* < 0.05 considered statistically significant. Statistical analysis was performed using Statistics Package for Social Science software version 17.0 for Windows.

## 3. Results

### 3.1. Preparation of Acellular Bovine Gallbladder Matrix

**Figure 1:**
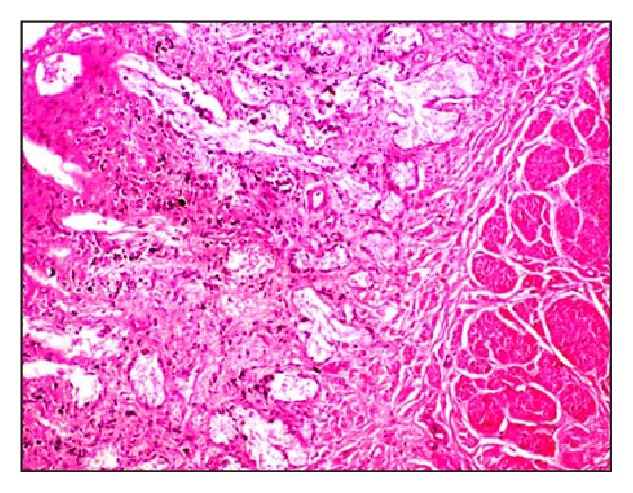
Microphotograph of native bovine gallbladder after delamination showing cellularity, loose muscular layer, and collagen fibers (H&E stain, 200x).

**Figure 2:**
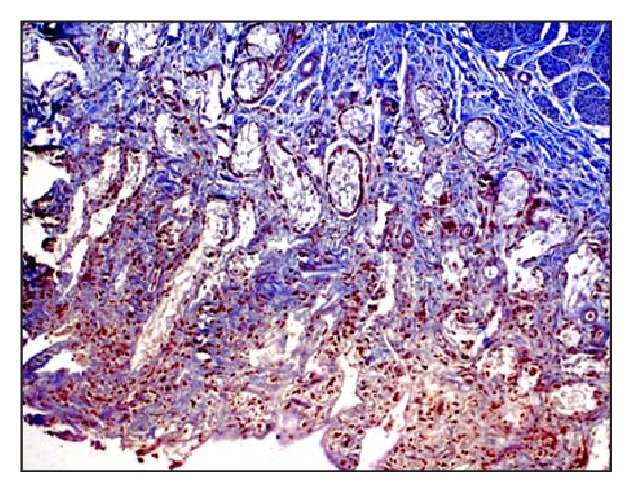
Microphotograph of native bovine gallbladder showing dense compact arrangement of collagen fibers (Masson's trichrome stain, 200x).

**Figure 3:**
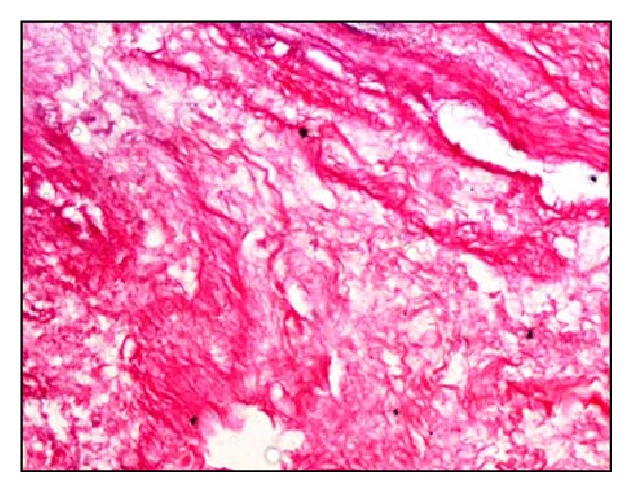
Microphotograph of acellular bovine gallbladder showing complete loss of cellularity (H&E stain, 200x).

**Figure 4:**
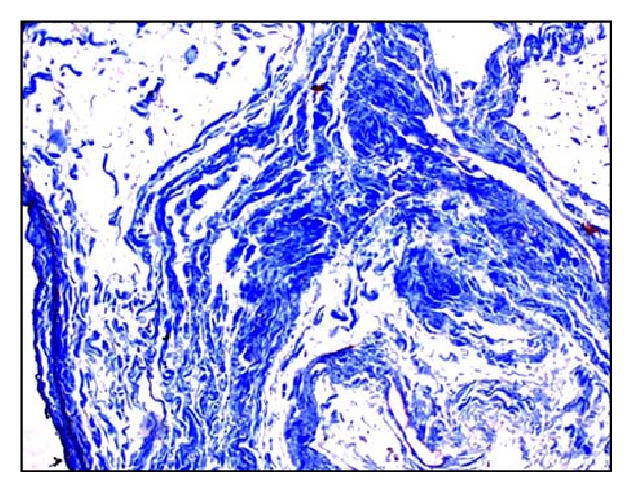
Microphotograph of acellular bovine gallbladder showing compact collagen fibers with moderate porosity than the native tissue (Masson's trichrome stain, 200x).

Microscopically, native bovine gallbladder after delamination showed cellularity, loose muscular layer, and collagen fibers (Figure 1). Masson's trichrome staining also showed dense compact arrangement of collagen fibers (Figure 2). The delaminated gallbladder treated with 0.5% ionic biological detergent for 12 h under constant agitation showed complete loss of cellularity. The submucosal layers were completely acellular (Figure 3). The collagen fibers were compact with moderate porosity than the native tissue (Figure 4).

### 3.2. Wound Contraction

Mean ± SE of the total wound area (mm^2^) of the skin wounds at different time intervals are presented in Table 1. A gradual decrease in wound area (mm^2^) was observed in all the groups during the entire observation period.

The wound area decreased significantly (*P* < 0.05) at various time intervals in different groups. However, the rate of decrease in wound area was faster in group III as compared to other groups, but there was no significant difference (*P* > 0.05) between groups III and I in later stages (day 28).

Percent contraction (mean ± SE) of wound area at different time intervals in different groups is presented in Table 2. A gradual increase in percent wound contraction was observed in all the groups during the observation period. Although the original wound area created was 2 × 2 cm^2^, the wounds were expanded to various extents in groups I and II because in group I no matrix was used and the wound healed as open wound and in group II commercially available collagen sheet was applied, and as it is very fragile in nature suturing was not possible. In group III prepared acellular cholecyst was sutured over the wound area to prevent drying and desiccation of the inner matrix. As the healing progressed, the wound area decreased significantly (*P* < 0.05) at different time intervals in all the groups.

A significant increase (*P* < 0.05) in percent contraction was observed in all groups during the observation period. On day 28, maximum wound contraction (98.92%) was recorded in group III animals where b-CEM was used.

### 3.3. Planimetry

**Figure 5:**
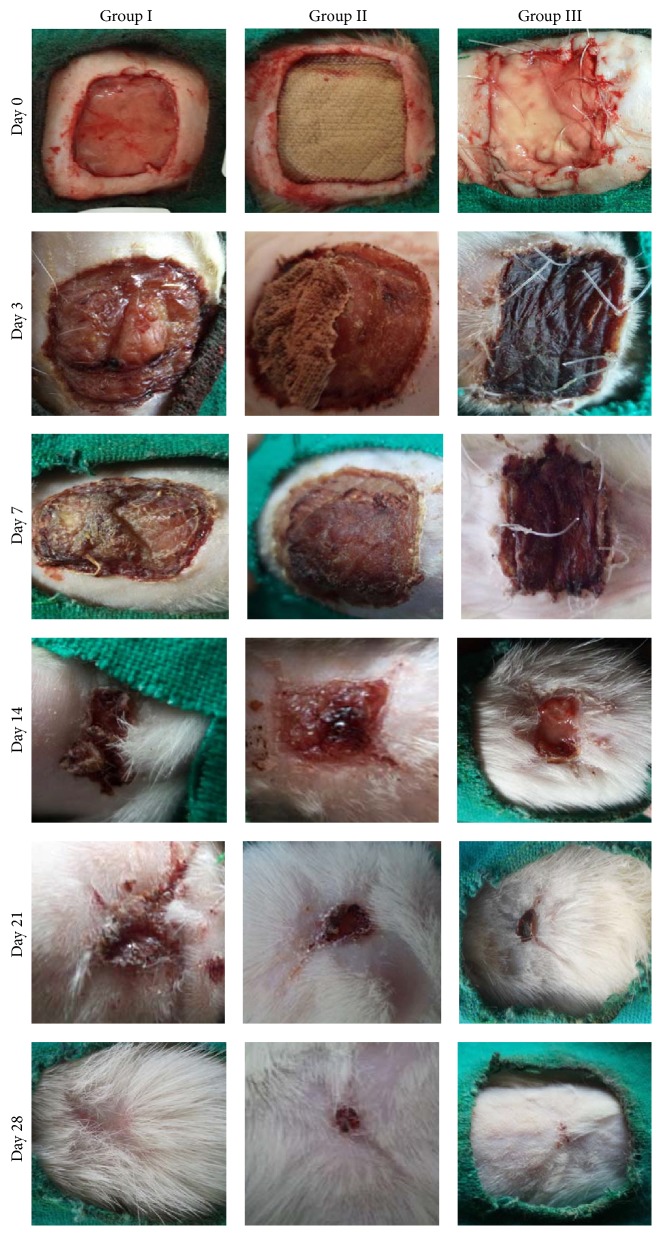
Digital colour photographs of different groups at different time intervals.

During the entire period of the study none of the wounds showed any visible suppurative inflammation. Furthermore, none of the animals became sick or died. The representative images of wound of one animal in each group, at baseline (day zero) and end of healing, are presented in Figure 5. On day 3, in control (I), wounds were covered with soft and fragile pinkish mass above the wound surface area with serous exudates oozing out. On day 7, the surface became more desiccated and necrosed with some amount of exudates. By day 14, the wound size decreased markedly and a thick crust developed and it starts detaching leaving a raw granular pink tissue. By day 28, wound healed up completely by severe contraction leaving a large scar. In b-CS(II), on day 3, the top layer of commercially collagen sheet implanted wound covered with soft and fragile pinkish mass necrosed margin with exudates. By day 7, the top layer of graft got dried and turned up brown. By day 14, the upper layer became more desiccated and shriveled. On day 28, the wound was not completely healed. In b-CEM on day 3, the top layer of acellular grafts appeared dark brown in colour. By day 7, it got dried and turned up brown. On day 14, it was seen in a stage of detachment from the underlying tissue as only the ends sutured holding it in place. On day 21, the dried-up top layer was completely sloughed off and newly formed granulation tissue within the under lying acellular graft covered the entire surface of the wound. By day 28, size of the wound decreased markedly. The wound edges healed completely and the remaining granulation tissue in the centre dried up indicating complete healing beneath it.

### 3.4. Haematological Observations

A significant (*P* < 0.05) increase in neutrophils percentage after operation was observed up to day 3 postoperatively in all the groups. Thereafter, they started decreasing and reached within normal limits on day 21. Animals of group I show significantly (*P* < 0.05) higher values on day 7, when compared to other groups. Thereafter, the values recorded were within the normal physiological range (10–30%). Contrary to increased neutrophil count, a significant (*P* < 0.05) decrease in lymphocyte count was observed up to day 7 in animals of all the groups postoperatively. The values fluctuated within the normal physiological range (65–85%) in various groups at different time intervals. Total leukocyte count was within normal physiological range (6–18%) in all groups at different time intervals.

### 3.5. Immunological Observations

The free protein contents of native bubaline gallbladder, decellularized bubaline gallbladder (b-CEM), and commercially available collagen sheet (b-CS) were estimated as per the methods of Lowry et al. [27] using bovine serum albumin (BSA) as a standard. The values of protein contents of native bubaline gallbladder, b-CEM, and b-CS are 3.76 mg/mL, 0.054 mg/mL, and 0.06 mg/mL, respectively.

#### 3.5.1. Humoral Response

The levels of antibodies present in serum prior to implantation were taken as basal values. The scaffolds specific antibodies were expressed as mean ± SE absorbance at 492 *η*m wavelength (OD_492_). ELISA reaction is presented in Table 3.

In control group no rise in antibody titer was observed as the wound healed open without any matrix. In the treatment groups II and III there was relative rise in antibody titer from day 0 to day 28 after implantation. The B-cell response was significantly higher (*P* < 0.05) in the groups II and III on day 28 as evidenced by higher absorbance values when compared with day 0 values. No significant difference (*P* > 0.05) existed between these treated groups.

#### 3.5.2. Cell Mediated Immune Response

Mean ± SE of stimulation index (SI) values of different groups on 28 days after implantation are presented in Table 4.

Against the collagen sheet, native BG antigen, the animals of group III showed significant amount of stimulation (*P* < 0.05). As compared to the SI values of Con A and PHA all the groups exhibited significant (*P* < 0.05) rise in SI values. The T-cell responses were lowest in the group III (implanted with acellular b-CEM) as evidenced by lower SI values.

### 3.6. Histological Observations

**Figure 6:**
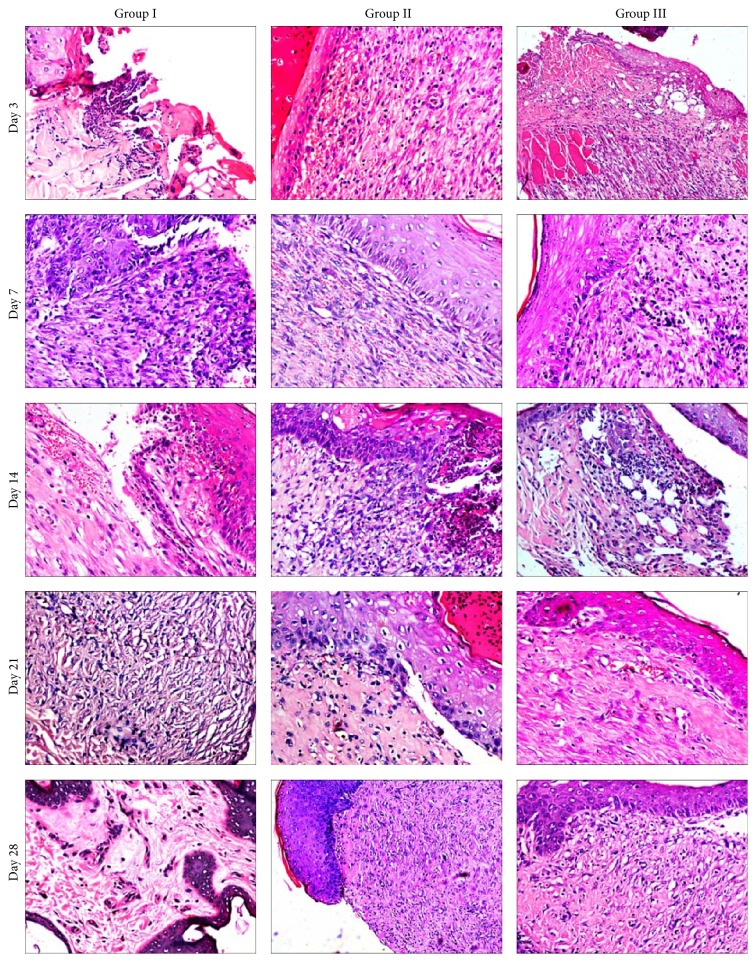
Microphotograph of different groups at different time intervals (H&E stain, 200x).

**Figure 7:**
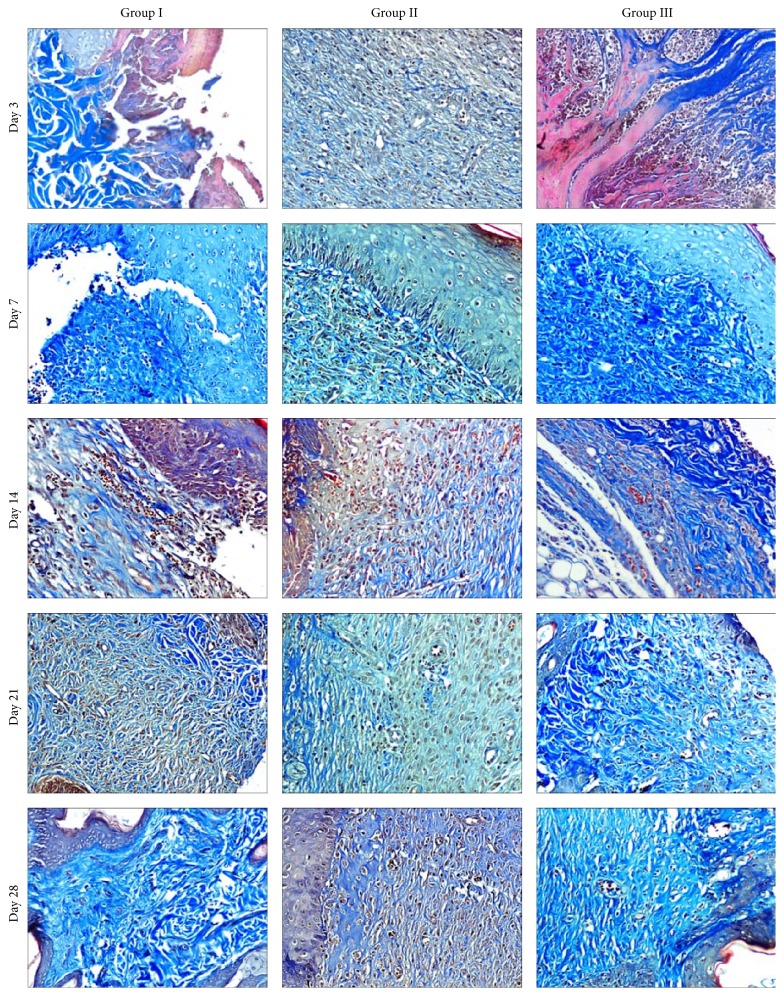
Microphotograph of different groups at different time intervals (Masson's trichrome stain, 200x).

The biopsy specimens from the implantation site were collected on days 3, 7, 14, 21, and 28 postoperatively. The samples were then processed for sectioning and later on hematoxylin and eosin staining. The results are presented in Table 5 and Figure 6. The samples were also subjected to Masson's trichrome staining for the appreciation of the collagen fibers formation and results are presented in Figure 7. The group having the least number of score was considered the best.

In control group (C) on day 3, the wound was covered with necrotic debris and admixed with high degree inflammatory cell infiltration, edema, and congestion. The underlying stoma contained few proliferated fibroblasts and some neovascularization along with spilled neutrophils. The collagen fibers were less dense, thin, and worst arranged. The total histopathological score was 27. On day 7, proliferation of fibroblasts and angiogenesis increases and no epithelialization was observed at the margin of wound area covering the granulation tissue. The granulation tissue (fibroblast and neovascularization) was very wide and had less dense, thin, and worst arranged collagen fibers and presence of moderate neutrophil and mononuclear cells (lymphocyte and macrophages). The histopathological score was 30 at this stage. On day 14, severe proliferation of fibroblasts and neovascularization were observed along with inflammatory cell infiltration and the score was 28. On day 21, collagen formation was evident in some areas (immature collagen) and inflammation was greatly reduced and the histopathological score was reduced to 23. On day 28, a high degree of collagen deposition with worst arrangement was observed. The score was 18 at this stage. On day 45, the surface epithelium was complete, but great contraction was seen in epithelium. The histopathological score was reduced to 13.

In group II (b-CS) inflammatory changes were observed on day 3 postoperatively. The fibroblast proliferation was found in collagen sheet implanted tissue and some neovascularization was also observed. There was necrosis and sloughing of the superficial layer and epithelialization was observed at edges of wound. The histopathological score was 26. On day 7, the wounded tissue nearer to host tissue was severely infiltrated by proliferating fibroblasts. The surface epithelium was thicker than normal skin. The fibrous tissue also revealed numerous blood vessels. The total histopathological score was 24. On day 14, the deeper layer of dermis revealed deposition of new collagen fibers in which fibroblasts were dispersed; epithelialization was incomplete with reduced inflammation. The score was 26 at this stage. By day 21, epithelialization had started. The inflammatory changes and neovascularization were reduced and score was reduced to 22. On day 28, superficial epithelialization was observed. The histopathological score was 16. The hair follicles and skin gland could also be observed on day 45 and epithelialization was complete with contraction. The total score was 11 at this stage.

In group III (b-CEM) on day 3, CEM graft was present over the healing wound tissue, but the neoepithelium was not prominent. Mild inflammatory changes were observed. The fibroblasts proliferation and neovascularization were observed in the graft tissue. The necrosis and sloughing of the superficial acellular scaffold matrix were observed and epithelialization was started at the edges of wound area. The collagen fibers were less dense, thin with worst arrangement. However, connections of graft collagen fibers with wound collagen fibers were observed. At this stage, the granulation tissue included only scattered collagen fibers and some newly formed blood vessels. The total histopathological score was 26. By day 7, fibroblast proliferation became more prominent and surface epithelium was thicker than normal skin. The superficial graft showed sloughing and underlying acellular matrix showed severe neovascularization within the stroma. The total score was 22. By day 14, inflammation and neovascularization were less as compared to day 7. The total histopathological score was 14. By day 21, epithelialization observed in margin was thicker than normal skin. The new collagen deposition in matrix was with best arrangement and the score was 14 at this stage. By day 28, the granulation tissue was covered and the epithelization was almost complete. The hair follicles and skin glands were also seen. The process of remodelling was evident on these samples with reorganization and disappearance of collagen; densely packed thick bundles were replaced by loosely placed thin strands. Total histopathological score was reduced to 12. On day 45, no contraction was seen on surface epithelium and the total histopathological score was 9.

## 4. Discussion

### 4.1. Wound Area and Percent Contraction

The wound contraction is the centripetal displacement of the wound edges that facilitates its closure after trauma. This process is carried out by myofibroblasts that contain *α*-actin from smooth muscle and is mediated by contractile forces produced by granulation tissue from wound [28]. The wound healing rate is defined as the gross epithelialization of the wound bed. The wound contraction was assessed by percent retention of the original wound area as reported by Schallberger et al. [14]. The wound contraction has been used to monitor wound healing. The wound area decreased gradually as the healing progressed. Maximum percent contraction was recorded in group I as no biomaterials were applied and wound healed as open wound. The wound in animals of this group healed completely by 28 days leaving a large scar indicating the existence of severe contraction. In group III the healing occurred with minimal contraction as the matrix was sutured with wound edges in this group and collagen sheet was simply applied over the wound area.

### 4.2. Haematological Observations

The neutrophils have been recognized as the first cellular defense of the body and neutrophilia along with leukocytosis is generally associated with inflammation [29, 30]. Besides the increase in neutrophil count, there was also decrease in lymphocyte count which returned near to baseline value by day 28 in all the animals of different groups. Moreover, DLC is proportional count of different WBCs where neutrophil and lymphocyte are major constituents. The DLC value revealed a significant increase in neutrophil percentage in early postoperative days in all the groups with gradual return nearer to normalcy, probably due to inflammatory response consequent to surgical trauma and foreign body reaction. Neutrophilia for short duration suggested the effect of surgical trauma rather than the implant provoked response. Similar observations were also reported by Gangwar et al. [31] following application of acellular dermal matrix for full thickness skin graft in rats.

The leukocyte counts have been used as an indicator to monitor progress of healing; the purpose of this study was to examine effects of graft on blood leukocyte numbers before an injury and during healing [32]. Total leukocyte count was within normal physiological range (6–18%) in all groups at different time intervals. In groups I and II it was elevated on days 3 and 7 as compared to group III.

### 4.3. Planimetry

Graft-assisted healing is an important strategy for treating full thickness skin wounds. The cholecyst derived scaffold was rich in natural biomolecules like elastin and glycosaminoglycans and when used as a xenograft, it promoted healing with excess cell proliferation at early phases and acceptable collagen deposition in the later remodelling phases [33]. After application of CEM matrix the colour of the implanted samples changed from white to dark brown and finally to dark, revealing that, on subsequent time intervals, decrease in vascularization and continuous loss of moisture contents of the matrix lead to changing its colour into black. Kaarthick [34] also reported change in matrix colour from white to brown and later on black colour after the repair of full thickness skin defects in rats. Upper matrix layer detached from the wound in form of scar and the underlying layer of b-CEM matrix was completely absorbed and newly formed granulation tissue within the matrix covered the whole surface of the wound. In group I (open wound) the healing was completed at day 28 with severe contracture and scarring as no matrix was used in this group. Minimum scar formation was observed in the remaining groups of animals.

### 4.4. Immunological Observations

The immunological response to biomaterial depends on the nature of processing and presence of potentially foreign antigen to various lymphocyte populations. The biomaterial could stimulate an antibody response, a cell mediated sensitization, or minimal to no response. The immune response to xenogeneic transplantation included both natural and induced humoral components [35].

The biological scaffolds are mainly composed of mammalian extracellular matrix and can be used for the reconstruction of various tissues and organs. These scaffolds are typically allogenic or xenogeneic in origin and derived from tissues such as gallbladder, dermis, pericardium, diaphragm, and small intestine submucosa. The cells in the ECM have class I and II histocompatibility antigens capable of eliciting rejection reactions. Also the cells have glycoproteins recognized by the immune system of hosts which elicit rejection reactions. Therefore, if these substances are eliminated from ECM, rejection reactions can be prevented. In the present study the animals of groups II and III showed slightly higher immune response on day 28 as compared to day 0. The least immune response was in animals of group I, where no graft was used. Slight immune response in groups II and III might be due to some immunogenic nature of decellularized ECM. The collagens are weakly immunogenic as compared to other proteins. However, complete elimination of alloantigens is considered difficult to perform and verify [36]. The acellular grafts were less immunogenic having better tolerance by allogenic hosts and equally effective as isograft [37]. The tissues were processed by decellularization and/or cross-linking to remove or mask antigenic epitopes and DNA [38]. The biologic scaffold processing methods plays an important role in determining the host response.

### 4.5. Histopathological Observations

Full thickness skin wounds are characterized by a complete destruction of the epithelial regenerative elements that reside in the dermis. Full thickness skin wound healing occurs by granulation tissue formation, contraction, and epithelialization [39]. The epithelialization occurs by migration of undamaged epidermal cells from the wound margins across the granulation bed [40]. The exogenous collagen supplementation enabled faster migration of cells that are involved in cutaneous wound healing. Since the exogenous collagen is molecular in nature [41] and supplies endogenous collagen* in vivo*, it readily integrates with the wound tissue and facilitates the attachment, migration, and proliferation of cells on the wound site [42].

The double acellular matrix applied in group III showed necrosis of the superficial matrix and underlying matrix showed severe fibroblastic proliferation with inflammatory cells. Group I exhibited severe proliferation of fibroblast with inflammatory cells in the wound area on day 3. Moderate degree of neovascularization was detected in all the groups. No new collagen formation was detected in any of the groups. But the graft implanted samples showed the existence of mature collagen of b-CEM origin. Although inflammation is necessary for healing by fighting infection and inducing the proliferation phase, healing proceeds only after inflammation is controlled [43]. During the initial stages of the wound healing, fibroblasts from the surrounding normal area are known to migrate and proliferate into the wound site and within 3-4 days get converted to myofibroblasts [44]. The main function of myofibroblasts in a healing wound is contraction by synthesizing of ECM proteins, notably, collagen types I–VI and XVIII, laminin, thrombospondin, glycoproteins, and proteoglycans for the dermal repair. The myofibroblasts usually go on increasing from the inflammatory stage (3-4 days). During the onset of healing, the proliferating fibroblast starts to synthesize collagen, and the total collagen content increases preferentially. On day 3, minimum score was observed in groups III and IV.

On day 7, after implantation, moderate to severe inflammation was present in all the groups and it was minimum in groups II and III. The early control of inflammation might be due to application of collagen matrix as in case of groups II and III it might facilitate the progress to the next phase of wound healing. The proliferation of fibroblasts was more in group III. Similar findings were observed by Perme et al. [45]. There was an increase in the extent of collagen deposition by 7 days (about 20%) and 14 days (about 40%) [33].

On day 14, the least scores were observed in groups III (21) followed by group II (26) which may be due to early acceptance of matrix. The sloughing of the upper layer of matrix was observed in all the groups except control group where wound remained open. It may be either due to the desiccation of the graft in high environmental temperature or due to an impaired formation of new blood vessels [46]. The myofibroblasts proliferate till the end of proliferation phase (14-15 days). Meanwhile, the underlying granulation tissue increased in mass that pushed up the graft upwards. The animals of groups II and III showed well-formed collagen and neovascularization with superficial epithelialization. The epithelialization and neovascularization were faster in group III as compared to other groups.

On day 21, the least score was observed in group III (14) followed by group II (22). The epithelialization was more similar to the normal skin in the wounds of these groups. In the remodelling phase (around 21–30 days), these myofibroblasts undergo apoptosis [47]. At 21 days of postimplantation the acellular matrix groups showed higher collagen synthesis as compared to the control group as appreciated by Masson's trichrome staining. Purohit [48] on day 21 found that the acellular dermal matrix throughout the width and length was replaced by mature collagenous connective tissue in experimentally created wounds in rabbits. On day 28, after implantation, the control group healed completely leaving abundant scar tissue but no complete healing was observed for other groups; also in group II contraction and scar formation were found. In group III collagen fiber arrangement was almost similar to normal skin. Marked fibroblastic response associated with an abundant new collagenous fibrous tissue was observed in these groups.

## Figures and Tables

**Table 1:** Mean ± SE of the wound area (mm^2^) at different time intervals in various groups.

Groups	Time intervals (days)					
	0	3	7	14	21	28
Group I (C)	429.22 ± 17.71	446.56 ± 9.12^B^	341.44 ± 14.82^B*∗*^	157.22 ± 10.97^AB*∗*^	45 ± 5.89^A*∗*^	5.56 ± 2.42^AB*∗*^

Group II (b-CS)	417.78 ± 18.94	386 ± 20.66^AB*∗*^	314.11 ± 22.50^AB*∗*^	131.78 ± 25.61^AB*∗*^	45.89 ± 8.44^A*∗*^	15.89 ± 6.55^B*∗*^

Group III (b-CEM)	444.67 ± 14.94	372.22 ± 26.26^A*∗*^	279.56 ± 16.61^AB*∗*^	104.44 ± 13.55^A*∗*^	23 ± 3.841^A*∗*^	4.44 ± 1.86^AB*∗*^

^AB^Values with different alphabets differ significantly (*P* < 0.05) between the groups at particular time intervals.

*∗* differ significantly (*P* < 0.05) from day 0 values.

**Table 2:** Mean ± SE of percent contraction of wound (%) at different time intervals in various groups.

Groups	Time interval (days)				
	3	7	14	21	28
Group I (C)	−4.88 ± 2.93^A^	70.76 ± 9.92^B^	62.96 ± 3.08^AB^	89.46 ± 1.32^A^	98.77 ± 0.47^B^

Group II (b-CS)	7.85 ± 1.76^B^	49.18 ± 12.36^AB^	72.75 ± 3.82^BC^	88.68 ± 2.28^A^	95.89 ± 1.87^A^

Group III (b-CEM)	16.48 ± 4.68^B^	48.30 ± 5.69^AB^	76.62 ± 3.06^C^	94.92 ± 0.76^B^	98.92 ± 0.47^B^

^ABC^Values with different alphabets differ significantly (*P* < 0.05) between the groups at particular time intervals.

**Table 3:** Mean ± SE of absorbance at 492 *η*m wavelength (OD_492_) of ELISA reaction on days 0 and 28.

Days	I (control)	II (b-CS)	III (b-CEM)
Day 0	0.23 ± 0.01	0.24 ± 0.00	0.28 ± 0.03
Day 28	0.22 ± 0.03^A^	0.38 ± 0.01^B^	0.35 ± 0.01^B^

^AB^Values with different alphabets differ significantly (*P* < 0.05) between the groups at particular time intervals.

**Table 4:** Mean ± SE of stimulation index (SI) on stimulation with collagen sheet, native BG gallbladder antigen, PHA, and ConA in various groups.

Groups	Ag	PHA	ConA
Group I (C)	0.28 ± 0.07	1.13 ± 0.22^*∗*^	1.22 ± 0.20^*∗*^
Group II (b-CS)	0.22 ± 0.02	1.21 ± 0.21^*∗*^	1.28 ± 0.19^*∗*^
Group III (b-CEM)	0.29 ± 0.03^A^	1.35 ± 0.16^B*∗*^	1.02 ± 0.05^B*∗*^

^AB^Values with different alphabets differ significantly (*P* < 0.05) between the groups at particular time intervals.

*∗* differ significantly (*P* < 0.05) from values of Ag.

**Table 5:** Histopathological scores of various treatment groups at different time intervals.

Parameters	Group I (c)						Group II (b-CS)						Group III (b-CEM)					
	Days																	
	3	7	14	21	28	45	3	7	14	21	28	45	3	7	14	21	28	45
Surface epithelium	4	4	3	2	2	2	3	2	2	2	2	1	3	2	2	2	1	1
Thickness of epithelium	1	2	4	3	2	1	2	4	4	4	3	1	2	4	3	2	2	1
Granulation tissue width	2	4	3	3	2	1	3	3	3	2	1	1	3	3	3	1	1	1
Inflammation	3	2	2	1	1	1	2	1	2	1	1	1	2	1	1	2	1	1
Fibroblast proliferation	2	4	3	4	2	1	4	3	4	3	2	1	4	4	3	2	2	1
Neovascularization	2	4	3	3	2	1	3	3	3	2	1	1	3	3	3	1	1	1
Collagen fiber density	3	3	3	2	1	1	3	2	2	2	1	1	3	1	2	1	1	1
Collagen fiber thickness	3	3	3	3	2	2	3	3	3	3	2	2	3	2	2	1	1	1
Collagen fiber arrangement	4	4	4	2	4	3	3	3	3	3	3	2	3	2	2	2	2	1
Total score	**27**	**30**	**28**	**23**	**18**	**13**	**26**	**24**	**26**	**22**	**16**	**11**	**26**	**22**	**21**	**14**	**12**	**9**

Surface epithelium: 1—complete, 2—incomplete, 3—initialization, and 4—absent.

Thickness of surface epithelium: 1—resembling normal skin, 2—slightly thick, 3—moderately thick, and 4—thicker than normal skin.

Granulation tissue width: 1—narrow, 2—slightly wide, 3—moderately wide, and 4—wide.

Inflammation: 1—absent, 2—mild, 3—moderate, and 4—severe.

Fibroblast proliferation: 1—resembling normal skin, 2—mild, 3—moderate, and 4—severe.

Neovascularization: 1—resembling normal skin, 2—mild, 3—moderate, and 4—severe.

Collagen fiber density: 1—denser, 2—dense, and 3—less dense.

Collagen fiber thickness: 1—thicker, 2—thick, and 3—thin.

Collagen fiber arrangement: 1—best, 2—better, 3—worse, and 4—worst.
